# Using the medication adherence reasons scale (MAR-scale) to identify the reasons for non-adherence in Chinese hypertensive patients

**DOI:** 10.1371/journal.pone.0325004

**Published:** 2025-06-10

**Authors:** Jingjing Pan, Bin Hu, Xiaorong Xue, Lian Wu

**Affiliations:** 1 Department of Pharmacy, Xi’an People’s Hospital (Xi’an Fourth Hospital), Xi’an, People’s Republic of China; 2 Department of Ophthalmology, Xi’an People’s Hospital (Xi’an Fourth Hospital), Xi’an, People’s Republic of China; Tribhuvan University Institute of Medicine, NEPAL

## Abstract

**Objectives:**

This study aimed to determine the prevalence of medication non-adherence and to explore the factors influencing it among Chinese hypertensive patients.

**Patients and methods:**

A total of 571 hypertensive patients hospitalized in a tertiary hospital in Xi’an, China were invited to participate in this cross-sectional study. The Chinese Version of Medication Adherence Reasons Scale (ChMAR-Scale) was used to identify the most common reasons for non-adherence to hypertension medications.Binary logistic regression analysis was employed to analyze independent risk factors for adherence in hypertensive patients.Descriptive statistics were used to calculate the adherence rates and trends in the reasons for non-adherence.

**Results:**

Approximately 66.9% of the patients did not adhere to their medications.Age (adjusted odds ratio [AOR] = 0.976, 95%CI:0.955–0.998, *P* = 0.032),education level(AOR = 0.566, 95% CI:0.419–0.765,*P* < 0.001) and blood pressure (BP) categories (AOR = 0.580, 95% CI: 0.439–0.767,*P* < 0.001) were independently associated with hypertensive medication adherence. Belief issues and self-perception issues were identified as the main reasons for medication non-adherence.These included self-adjustment of medications according to BP or physical condition, checking whether the medicine was still needed, concerns about long-term effects, and the belief that there was no longer a need to take the medicine.

**Conclusion:**

Poor medication adherence is widespread among Chinese hypertension patients. More attention should be paid,and effective strategies should be developed to address the factors affecting treatment adherence.These factors include certain sociodemographic factors,such as age,education level and BP categories as well as belief issues and self-perception issues of hypertensive patients. The findings of this study can potentially assist healthcare providers in formulating targeted interventions to improve medication adherence.

## Introduction

Hypertension (HTN) ranks among the most challenging public health issues globally. It is generally regarded as the primary cause of cardiovascular diseases [1], strokes [2] and chronic kidney failure [3]. According to the World Health Organization (WHO), 1.13 billion individuals are affected globally by hypertension [4]. Moreover, it is estimated to affect one-third of the world’s population by 2025 [5].Although the prevalence of HTN has seen a slight decline in some countries,its control rates remain disappointingly low [6,7]. For instance,in 2018, the prevalence of hypertension among adults in United States was approximately 40.58%, yet the control rate only reached 51.71% [8]. In China,a report indicated that the prevalence of hypertension among adults aged 18–69 years was 24.7%,with a meager control rate of just 12.0% [9]

Non-adherence to treatment is widely acknowledged as a major contributor to the low control rate of hypertension [10]. Adherence has been defined as the extent to which a person’s behavior-taking medication, following a diet, and/or executing lifestyle changes corresponds with agreed recommendations from a health care provider [11]. Therefore, identifying the root causes of non-adherence and implementing targeted interventions are crucial steps in enhancing treatment adherence.

Several methods for measuring adherence have been developed,which can be classified into direct and indirect methods. Direct methods include mobile health technologies, electronic medication monitoring systems,and biological assays etc [12–14]. Indirect methods mainly include self-reported questionnaires and scales. Compared to direct methods, indirect methods are more commonly used due to their economy and availability. Nevertheless, the most frequently used non-adherence questionnaires, like the Morisky scale and Hill-Bone scale,are based on the classification of non-adherence as intentional and unintentional. They offer only a limited number of potential reasons for non-adherence, thus making it difﬁcult to formulate targeted intervention [15,16].

To overcome the limitations of previous scales, Unni and Farris developed the Medication Adherence Reasons Scale (MAR-Scale).This scale encompasses a comprehensive set of reasons for non-adherence,which are grouped into four domains: believe, forgetfulness, management, and availability [17,18]. The MAR-Scale with its 19 commonly reported non-adherence reasons,can detect non-adherence more comprehensively.Subsequently,Chen et al.translated the MAR-Scale into Chinese and developed the Chinese Version of Medication Adherence Reasons Scale (ChMAR-Scale) [19].

Although certain Chinese-language scales have been used to assess medication adherence in hypertensive, they are restricted in discerning the underlying causes of non-adherent behaviors [20,21]. The objective of this study was to determine the prevalence and associated factors of medication non-adherence. More significantly, it aimed to explore the potential reasons for non-adherence among hypertensive patients by using the ChMAR-Scale and to develop corresponding intervention strategies.

## Participants and methods

This study was a hospital-based, cross-sectional survey conducted in Xi’an People’s Hospital (Xi’an Fourth Hospital) in Xi’an, China between February and May 2024. Patients were invited to take part in the study if they met the following inclusion criteria: (1) ≥18 years-old, (2) diagnosis of primary hypertension, (3) undergoing antihypertensive drug therapy, and (4) agreeing to participate in the study. The exclusion criteria for all patients included:(1) severe complications of hypertension (e.g., severe heart failure and stroke), (2) could not communicate due to physical or mental problems, and (3) pregnant women. Finally, 571 hypertensive patients were randomly selected and were recruited in this study.

The study was approved by the ethics committee of school of medicine, Xi’an Jiaotong University. Written informed consent from all participants was obtained and all procedures were performed in accordance with the Declaration of Helsinki and relevant policies in China. Investigators explained the goal of the study and gave the standardized instructions before they distributed questionnaires to patients. The patients were asked to complete the questionnaires independently.Patients who could not read Chinese were assisted by investigators by reading each item for them to facilitate questionnaire complication.

Patients’ data referring to sociodemographic factors including gender, age, occupational status, health insurance, education level, smoking status and part of clinical data such as BP categories were collected by reviewing the electronic medical records.Individuals were categorized as smokers if they were current smokers.Some clinical data including duration of HTN, duration of antihypertensive drugs used and the number of antihypertensive drugs used which could not be obtained from the electronic medical records were collected through interview by investigators.

HTN adherence and the reasons for non-adherence was measured using the ChMAR-Scale. The ChMAR-Scale questionnaire consists 24 items and one global item. The global item provides an overall estimate of the frequency of medication adherence, while the remaining 24 items measuring specific reasons for non-adherence. Subjects were asked, “Over the last 30 days, how often were you able to take your blood pressure medicine exactly as prescribed?” The response was on a 5-point scale, ranging from 1 (all the time) to 5 (none of the time). If the respondent selected any option other than “all the time”, they are considered non-adherent based on the global item.Subsequently,then they were asked to indicate how often they were non-adherent with their antihypertensive medications for each of the reasons using a 5-point scale ranging from 1 (all the time) to 5 (none of the time). Participants were classified as non-adherent if they did not response “none of the time” to one or more items among the 24 items of the scale. The scale consists of six subgroups: subgroup 1: belief issues(8 items), subgroup 2: self-perception issues (3 items),subgroup 3: forgetfulness issue(3 items), subgroup 4:managing issues (4 items), subgroup 5:availability issues(3 items) and subgroup 6: miscellaneous(3 items).The ChMAR-Scale had acceptable Cronbach’s alpha values, ranging from 0.649 to 0.852.The factor loading for each item was above acceptable levels and ranged from 0.365 to 0.775 [19].

## Data analysis

Descriptive statistics were employed to analyze the socio-demographic data and clinical characteristics of hypertensive patients.Binary logistic regression analysis was used to identify the independent risk factors for adherence of hypertensive patients.Descriptive statistics were also applied to calculate adherence rates and trends in the reasons for non-adherence among all participants. A significance level of *P* < 0.05 was regarded as statistically significant. All statistical analyses were performed using SPSS version 19 (IBM Corp., Armonk, NY, USA).

## Results

Among the 571 participants, 293 (51.31%) were male, and over half (56.74%) were 65 years old or older.A total of 346(60.60%) participants had retired, and the vast majority (89.32%) had medical insurance. 423(74.08%) patients were from urban areas, while 148 (25.92%) were from rural areas.Only 7 patients (1.23%) were illiterate, and 157 (27.50%) patients had a college or university degree.The majority (88.09%) of the patients did not smoke.Nearly half of the patients (48.51%) had been diagnosed with HTN within the past 10 years, and more than half (52.54%) were diagnosed with stage III HTN.Approximately 31.00% of the participants had been prescribed antihypertensive drugs for less than 5 years, and 17.51% had been on such medications for more than 20 years. Most patients (76.88%) took only one antihypertensive medicine daily,while 13 (2.28%) patients took more than 3 antihypertensive medicines daily (Table 1).

**Table 1:** Sociodemographic and clinical characteristics of hypertension patients (n = 571).

Characteristics	Frequency (n)	Percentage(%)
**Gender**		
Male	293	51.31
Female	278	48.69
**Age**		
<45	12	2.10
45-65	235	41.16
65-80	263	46.06
≥80	61	10.68
**Occupation**		
Retired	346	60.60
Unemployed	25	4.38
Peasant farmer	134	23.47
Employed	66	11.56
**Health insurance**		
Employee medical insurance	19	3.33
Urban resident medical insurance	491	85.99
No medical insurance	61	10.68
**Residence**		
Urban	423	74.08
Rural	148	25.92
**Education level**		
Illiteracy	7	1.23
Primary	126	22.07
High school	281	49.21
College/University	157	27.50
**Current smoking status**		
Not smoking	503	88.09
Smoking	68	11.91
**Duration of HTN (years)**		
<5	177	31.00
5-10	100	17.51
10-20	177	31.00
≥20	117	20.49
**BP categories**		
Grade I Hypertension	75	13.13
Grade II Hypertension	196	34.33
Grade III Hypertension	300	52.54
**Duration of antihypertensive drugs used (years)**		
<5	177	31.00
5-10	102	17.86
10-20	192	33.63
≥20	100	17.51
**Number of antihypertensive drugs used**		
1	439	76.88
2	119	20.84
≥3	13	2.28

**Fig 1:**
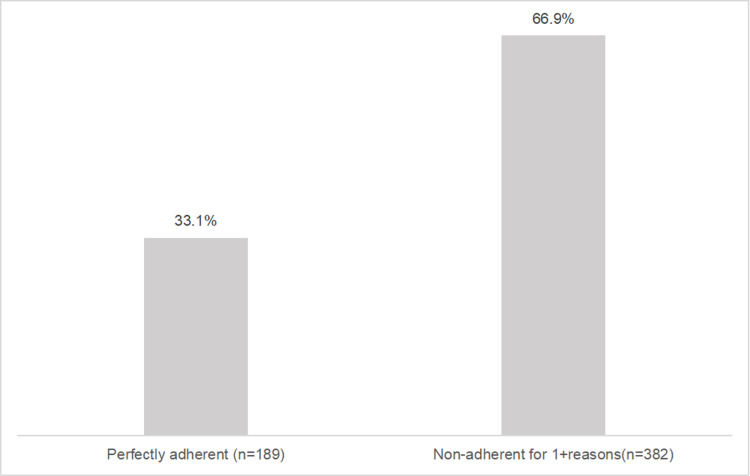
Participant adherence distribution(n ** = 571).** Total sample:571 participants,with 189(33.1%)demonstrating good adherence and 382(66.9%) showing poor adherence.

Among the 571 participants, 189 patients (33.1%)exhibited satisfactory adherence to antihypertensive treatment,whereas the remaining 382 (66.9%) patients were non-adherent(Fig 1).

Three factors were found to have an independent association with antihypertensive treatment adherence:age (*P* = 0.032), education level(*P* < 0.001) and blood pressure categories (*P* < 0.001). Specifically, drug adherence increased as the age of the hypertensive patients grew (*P* = 0.032, AOR = 0.976, 95% CI: 0.955–0.998).Moreover,with an increase in the education level of hypertensive patients, their medication adherence also rose accordingly (*P* < 0.001, AOR = 0.566, 95% CI: 0.419–0.765). In this study,it was observed that the lower the category of hypertension,the greater the adherence of hypertensive patients (*P* < 0.001, AOR = 0.580, 95% CI: 0.439–0.767)(Table 2).

**Table 2:** Binary logistic regression analysis of factors associated with medication non-adherence.

Variables	COR (95% Cl)	*P*-value	AOR (95% Cl)	*P*-value
**Age**	0.979(0.963-0.996)	0.015	0.976(0.955-0.998)	0.032
**Education level**	0.685(0.537-0.874)	0.002	0.566(0.419-0.765)	<0.001
**BP categories**	0.774(0.607-0.987)	0.039	0.580(0.439-0.767)	<0.001

**COR**, crude odds ratio**; AOR**, adjusted odds ratio; **Cl**, confidence interval; ***P***, probability.

**Fig 2:**
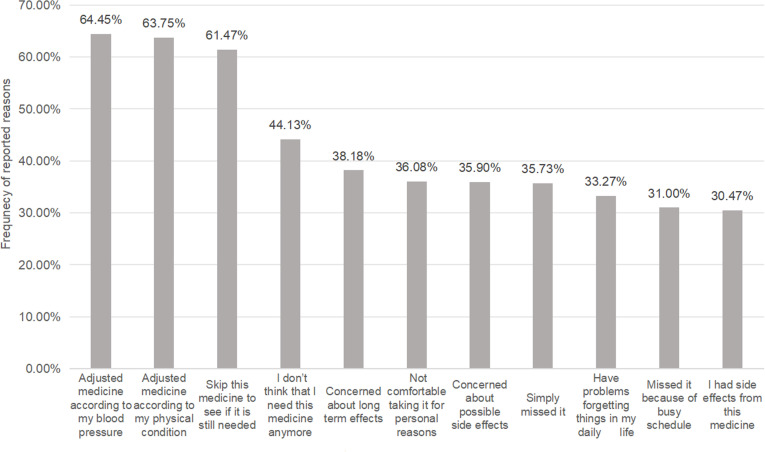
Top reasons for medication non-adherence(n ** = 571).** The top 11 reasons for medication non-adherence (ranked highest to lowest),each contributing to >30% of cases among 571 participants.

The average scores according to the ChMAR-Scale, as well as the percentage and distribution for each reason of medication non-adherence in each item,were presented in Table 3. The top eleven reasons (with a proportion>30%) were displayed in Fig 2.The two most common reasons were “I adjusted medicine according to my blood pressure” and“ I adjusted medicine according to my physical condition”, with 64.45% and 63.75% of the participants reporting these reasons respectively. Next was “I sometimes skip this medicine to see if it is still needed”,accounted for 61.47% of the participants. “I don’t think that I need this medicine anymore” (44.13%) and “ I am concerned about long term effects from this medicine” (38.18%) ranked as the fourth and fifth most common reasons respectively.Only a tiny proportion of the respondents missed taking their medications due to reasons like not having money to pay for the medicine(0.18%) and having difficulty swallowing the medication(0.35%).Notably, no one reported “having difficulty opening the container” as a reason for non-adherence.

**Table 3:** The frequency and percentage of reasons for medication non-adherence.

Reasons for Nonadherence Items	M ± SD	Never, n(%)	A Little of the time, n(%)	Sometimes, n(%)	Mostly, n(%)	All the Time, n(%)	Non-Adherence (%)
**Factor 1: Belief issues**							
Q1. I am concerned about possible side effects from this medicine	4.44 ± 0.84	366(64.10%)	112(19.61%)	73(12.78%)	20(3.50%)	0(0)	35.90%
Q2. I am concerned about long term effects from this medicine	4.38 ± 0.91	353(61.82%)	108(18.91%)	84(14.71%)	24(4.20%)	2(0.35%)	38.18%
Q3. I sometimes skip this medicine to see if it is still needed	3.54 ± 1.40	220(38.53%)	88(15.41%)	92(16.11%)	122(21.37%)	49(8.58%)	61.47%
Q4. I had side effects from this medicine	4.58 ± 0.71	397(69.53%)	114(19.96%)	53(9.28%)	7(1.23%)	0(0)	30.47%
Q5. I was not comfortable taking it for social reasons (e.g., I was with friends)	4.78 ± 0.54	475(83.19%)	71(12.43%)	21(3.68%)	4(0.70%)	0(0)	16.81%
Q6. I don’t think that this medicine is working for me	4.95 ± 0.27	551(96.50%)	14(2.45%)	5(0.88%)	1(0.02%)	0(0)	3.50%
Q7. I was not comfortable taking it for personal reasons (e.g., I was travelling)	4.42 ± 0.88	365(63.92%)	106(18.56%)	78(13.66%)	19(3.33%)	3(0.53%)	36.08%
Q8. I do not consider taking this medicine as a high priority in my daily routine	4.90 ± 0.41	528(92.47%)	34(5.95%)	5(0.88%)	2(0.35%)	2(0.35%)	7.53%
**Factor 2: Self- perception issues**							
Q9. I adjusted medicine according to my physical condition	3.54 ± 1.35	207(36.25%)	92(16.11%)	120(21.02%)	109(19.09%)	43(7.53%)	63.75%
Q10. I adjusted medicine according to my blood pressure	3.52 ± 1.37	203(35.55%)	93(16.29%)	120(21.02%)	103(18.04%)	52(9.11%)	64.45%
Q11. I don’t think that I need this medicine anymore	4.33 ± 0.90	319(55.87%)	146(25.57%)	84(14.71%)	14(2.45%)	8(1.40%)	44.13%
**Factor 3: Forgetfulness**							
Q12. I would have taken it but simply missed it	4.49 ± 0.78	367(64.27%)	129(22.59%)	62(10.86%)	13(2.28%)	0(0)	35.73%
Q13. I would have taken it but have problems forgetting things in my daily life	4.51 ± 0.78	381(66.73%)	116(20.32%)	60(10.51%)	14(2.45%)	0(0)	33.27%
Q14. I would have taken it but missed it because of busy schedule	4.56 ± 0.56	394(69.00%)	114(19.96%)	50(8.76%)	12(2.10%)	1(0.18%)	31.00%
**Factor 4: Managing issues**							
Q15. I have trouble managing all the medicines I have to take	4.71 ± 0.62	453(79.33%)	77(13.49%)	37(6.48%)	3(0.53%)	1(0.18%)	20.67%
Q16. I am not sure how to take this medicine	4.99 ± 1.44	565(98.95%)	4(0.70%)	2(0.35%)	0(0)	0(0)	1.05%
Q17. I had difficulty opening the container	5.00 ± 0.00	571(100%)	0(0)	0(0)	0(0)	0(0)	0
Q18. I had difficulty swallowing this medicine	5.00 ± 0.06	569(99.65%)	2(0.35%)	0(0)	0(0)	0(0)	0.35%
**Factor 5: Availability issues**							
Q19. I didn’t have the medicine because I didn’t have a ride to the pharmacy	4.81 ± 0.56	500(87.57%)	46(8.06%)	17(2.98%)	6(1.05%)	2(0.35%)	12.43%
Q20. I didn’t have the medicine because I didn’t have time to go to the pharmacy	4.93 ± 0.31	542(94.92%)	21(3.68%)	7(1.23%)	1(0.18%)	0(0)	5.08%
Q21. I didn’t have the medicine because the pharmacy was out of this medicine	4.99 ± 0.14	566(99.12%)	3(0.53%)	2(0.35%)	0(0)	0(0)	0.88%
**Factor 6: Miscellaneous**							
Q22. I was also taking folk therapy so I adjusted the dosage of my blood pressure medicine	4.73 ± 0.63	463(81.09%)	68(11.91%)	32(5.60%)	8(1.40%)	0(0)	18.91%
Q23. I was also taking Chinese Medicine so I adjusted the dosage of my blood pressure medicine	4.55 ± 0.81	409(71.63%)	87(15.24%)	55(9.63%)	19(3.33%)	1(0.18%)	28.37%
Q24. I did not have money to pay for this medicine	5.00 ± 0.04	570(99.82%)	1(0.18%)	0(0)	0(0)	0(0)	0.18%

## Discussion

The study aimed to evaluate the degree of non-adherence, explored the influencing factors, and focus on the potential reasons for non-adherent behaviors in hypertensive patients. The results showed that only 189 patients (33.1%) adhered to their antihypertensive treatment,indicating that the adherence among hypertensive patients in China was unsatisfactory.There are several other investigations with findings similar to ours [20,22,23].The medication adherence rate observed in this study was notably lower than that reported in developed countries like the USA [24]and Germany [25],and it was also lower compared to some other developing countries [26,27]. Antihypertentive medication adherence is critical for hypertension control.Poor medication adherence has been reported as the most common cause of lack of response to pharmacological treatment [28].Improving medication adherence can significantly enhance the therapeutic effect, thereby greatly increasing the treatment outcome [29].However, our study revealed that poor medication adherence remains a clinical challenge in the management of hypertension in China.Consequently,Chinese healthcare providers should place greater emphasis on medication non-adherence among hypertensive patients to ensure that these patients can reach their healthcare objectives.

In this study, It was reported that the medication adherence increased as the age of the hypertensive patients rose. This finding was consistent with some previous studies [30,31]. One possible explanation is that as the hypertensive patients get older, they become more health-conscious.Moreover, most of them have retired and thus have more time to ensure regular medication intake. Another finding was that with an increase in the education level of hypertensive patients, their medication adherence also increased accordingly. It is quite understandable that patients with a higher education level posses more knowledge about the disease, its risks and the significance of adhering to medicine.The study also found that the lower the hypertension category, the greater the adherence among hypertensive patients.This results was similar to that of a previous study which reported that patients diagnosed with grade 3 hypertension were less adherent than those with grade 1 or grade 2 hypertension [22].A possible reason could be that patients with grade 3 hypertension generally have had hypertension for a long time,and some of them are worried about drug addiction or long-term side effects of antihypertensive drugs.

This study focused on the potential reasons of medication non-adherence in hypertensive patients. The ChMAR-Scale was used to identified the potential reasons of non-adherence behaviors. Despite being longer than some other scales, the ChMAR-Scale enables healthcare providers to gain a more profound understanding of why patients fails to take their medicines as prescribed.The 24 items related to reasons for non-adherence can serve for the purpose of intervention.Referred to the potential reasons, the most prevalent ones in this study were self-adjustment medications according to blood pressure or physical condition,skipping medications to check if they were still required, concerns about long-term effects, and no longer a need to take the medicine.It was showed that the main reasons for medication non-adherence were focused on two aspects: belief issues and self -perception issues.

In this study, the self-adjustment of medications based on BP or physical condition emerged as the primary reason for medication non-adherence.Several other studies have investigated the reasons of medication non-adherence and their top reasons vary. For instance,a study conducted in Singapore discovered that “fear of developing drug dependence” was the most prevalent reason for medication non-adherence [32]. Another study conducted in Malaysia [33] reported that five main categories such as the attitude towards drawback of western medication, and the poor relationship between healthcare providers and patients,contributed to medication non-adherence.However, according to most previous studies on the reasons for non-adherent behaviors, forgetfulness and concerns about side effects or long-term effects were the most common reasons [33–38].

In our study, belief issues and self perception issues were identified as barriers to medication adherence among Chinese hypertensive patients.These issues reflected patients’ lack of knowledge and negative beliefs about medications. They were insufficiently informed about the importance of taking medication and the potential medication side effects.Clinicians can engage in conversations with hypertensive patients to address and clarify their concerns regarding medications.Additionally, pharmacists can offer counseling and essential education about prescribed medications to enhance patients’ awareness of the significance of adhering to their treatment regimens. Meanwhile,the patient-provider relationship is considered as one of the facilitators for medication adherence [33]. Healthcare providers should improve their communication skills with patients and demonstrate more empathy towards patients’ concerns about their medication.

In our study,cost concern was a relatively minor factor contributing to non-adherence.This was mainly because the majority of patients had medical insurance, and most blood pressure medications were covered by it.No patient in this study reported having difficulty opening the container. Although opening containers is not a common issue for antihypertensive medicines, it is a significant reason for non-adherence in asthma medications [17].Since the ChMAR- Scale can be used to identify non-adherence reasons across multiple disease conditions, the item “difficulty opening containers”was not excluded in this scale.

This study had several limitations.The samples were conveniently selected from a restricted area in China with a relatively homogeneous population, so the findings may not be applicable to a broader population.Additionally,the measurement of adherence relied on self-reported questionnaires, which could potentially introduce recall bias.Moreover, the sample size was small. Larger scale investigations should be carried out to gain more comprehensive insights in the future.

## Conclusion

In this study, almost two-thirds of hypertensive participants did not adhere to their treatment regimens.Certain sociodemographic factors,including age,education level and BP categories, were found to be independently associated with antihypertensive treatment adherence. Additionally,belief issues and self-perception issues were recognized as prevalent potential reasons of non-adherence.This information can be valuable for healthcare providers.By understanding these individualized reasons for non-adherence,they can design targeted interventions tailored to each patient’s specific situation.Such interventions have the potential to significantly improve treatment adherence among hypertensive patients,ultimately leading to better management of the condition and improved patient outcomes.

## Supporting information

S1 Data All raw data required to replicate the results of the study. (XLSX)

S1 File The chinese version of medication adherence reasons scale. (DOCX)
