# Supplementary material for: Using the medication adherence reasons scale (MAR-scale) to identify the reasons for non-adherence in Chinese hypertensive patients
Source: PLoS One. 2025 Jun 10;20(6):e0325004. doi: 10.1371/journal.pone.0325004 (PMC12151354; doi:10.1371/journal.pone.0325004)
Supplement: S1 File — (DOCX) [file pone.0325004.s002.docx]

**The Chinese Version of Medication Adherence Reasons Scale**

| Q1. I am concerned about possible side effects from this medicine |
| --- |
| Q2. I am concerned about long term effects from this medicine |
| Q3. I sometimes skip this medicine to see if it is still needed |
| Q4. I had side effects from this medicine |
| Q5. I was not comfortable taking it for social reasons (e.g., I was with friends) |
| Q6. I don’t think that this medicine is working for me |
| Q7. I was not comfortable taking it for personal reasons (e.g., I was travelling) |
| Q8. I do not consider taking this medicine as a high priority in my daily routine |
| Q9. I adjusted medicine according to my physical condition |
| Q10. I adjusted medicine according to my blood pressure |
| Q11. I don’t think that I need this medicine anymore |
| Q12. I would have taken it but simply missed it |
| Q13. I would have taken it but have problems forgetting things in my daily life |
| Q14. I would have taken it but missed it because of busy schedule |
| Q15. I have trouble managing all the medicines I have to take |
| Q16. I am not sure how to take this medicine |
| Q17. I had difficulty opening the container |
| Q18. I had difficulty swallowing this medicine |
| Q19. I didn’t have the medicine because I didn’t have a ride to the pharmacy |
| Q20. I didn’t have the medicine because I didn’t have time to go to the pharmacy |
| Q21. I didn’t have the medicine because the pharmacy was out of this medicine |
| Q22. I was also taking folk therapy so I adjusted the dosage of my blood pressure medicine |
| Q23. I was also taking Chinese Medicine so I adjusted the dosage of my blood pressure medicine |
| Q24. I did not have money to pay for this medicine |
